# Dynamic habitat corridors for marine predators; intensive use of a coastal channel by harbour seals is modulated by tidal currents

**DOI:** 10.1007/s00265-016-2219-7

**Published:** 2016-10-14

**Authors:** Gordon D. Hastie, Deborah J.F. Russell, Steven Benjamins, Simon Moss, Ben Wilson, Dave Thompson

**Affiliations:** 1 Sea Mammal Research Unit, Scottish Oceans Institute, University of St Andrews, St Andrews, Fife, KY16 8LB UK; 2 Scottish Association for Marine Science, University of the Highlands and Islands, Scottish Marine Institute, Oban, Argyll PA37 1QA UK; 3 Centre for Research into Ecological and Environmental Modelling, The Observatory, University of St Andrews, Fife, Scotland KY16 9LZ UK

**Keywords:** Foraging, Marine mammal, Oceanographic, Predator, Pinniped, Diving

## Abstract

**Abstract:**

Previous studies have found that predators utilise habitat corridors to ambush prey moving through them. In the marine environment, coastal channels effectively act as habitat corridors for prey movements, and sightings of predators in such areas suggest that they may target these for foraging. Unlike terrestrial systems where the underlying habitat structure is generally static, corridors in marine systems are in episodic flux due to water movements created by tidal processes. Although these hydrographic features can be highly complex, there is generally a predictable underlying cyclic tidal pattern to their structure. For marine predators that must find prey that is often patchy and widely distributed, the underlying temporal predictability in potential foraging opportunities in marine corridors may be important drivers in their use. Here, we used data from land-based sightings and 19 harbour seals (*Phoca vitulina*) tagged with high-resolution GPS telemetry to investigate the spatial and temporal distribution patterns of seals in a narrow tidal channel. These seals showed a striking pattern in their distribution; all seals spent a high proportion of their time around the narrowest point of the channel. There was also a distinctive tidal pattern in the use of the channel; sightings of seals in the water peaked during the flood tide and were at a minimum during the ebb tide. This pattern is likely to be related to prey availability and/or foraging efficiency driven by the underlying tidal pattern in the water movements through the channel.

**Significance Statement:**

To maximise foraging efficiency, predators often make use of narrow constrictions in habitat to intercept prey using these corridors for movement. In the marine environment, narrow channels may act as corridors, and sightings of predators suggest that they may target these for foraging. Despite this, there is little information on how individual predators use such areas. Here, we investigate how individual harbour seals use a narrow coastal channel subject to strong tidal currents; results showed that seals spent the majority of their time at the narrowest point of the channel foraging during peak tidal currents. This highlights the importance of narrow channels for marine predators and suggests that this usually wide-ranging predator may restrict its geographic range to forage in the channel as a result of increased prey availability and/or foraging efficiency driven by water movements through the narrow corridor.

**Electronic supplementary material:**

The online version of this article (doi:10.1007/s00265-016-2219-7) contains supplementary material, which is available to authorized users.

## Introduction

The distribution patterns exhibited by predators are primarily shaped by the distribution of their prey, and to maximise foraging efficiency, predators appear to make use of habitat features to either increase prey encounter rates or to increase their prey capture efficiency (e.g. Kauffman et al. 2007). Corridors are narrow strips that connect disjunct patches of habitat (e.g. Rosenberg et al. 1997; Beier and Noss 1998; Haddad et al. 2003), and several studies have found that predators utilise such corridors to ambush prey that use them for regular movement (Brinkerhoff et al. 2005; Knowlton and Graham 2010). For example, brown bears (*Ursus arctos*) foraging on sockeye salmon (*Oncorhynchus nerka*) during salmon upstream migrations tend to focus on narrow, shallow parts of streams to catch fish; predation events in wider, deeper parts of streams are far lower (Gard 1971). Cod (*Gadus morhua*) and saithe (*Pollachius virens*) have also been shown to aggregate at small restricted areas of river mouths to feed on migrating salmon (*Salmo salar*) smolts (Hvidsten and Mokkelgjerd 1987; Hvidsten and Lund 1988).

In the marine environment, narrow coastal channels between land masses effectively act as corridors for movement of mobile marine species, and previous studies suggest that marine predators may target these for foraging. For example, bottlenose dolphin (*Tursiops truncatus*) sightings have been shown to be higher in narrow coastal channels compared to surrounding habitats (Wilson et al. 1997) and numbers of foraging events peaked in these constricted areas (Hastie et al. 2004). There is evidence to suggest that harbour seals (*Phoca vitulina*) may be attracted to narrow geographic constrictions (Brown and Mate 1983; Thompson et al. 1991; Suryan and Harvey 1998; Zamon 2001). Observations of harbour seals in the Moray Firth (Scotland) showed that a narrow channel was routinely used as a feeding area by up to 44 seals (Thompson et al. 1991). Brown and Mate (1983) also reported harbour seals foraging on salmon during the incoming tide at a constriction in Netarts Bay (Oregon, USA). In a tidal strait in San Juan Islands, Washington State, Zamon (2001) studied the spatial patterns of Pacific harbour seals (*P. v. richardsi*) in a coastal channel and showed that seals aggregated near the most constricted part of the channel.

Unlike terrestrial systems where the underlying habitat structure associated with corridors is generally relatively static, corridors in marine systems are in constant flux due to water movements created by tidal and meteorological processes. This can be particularly apparent for narrow channels that are often hydrographically dynamic with high current velocities (Wolanski and Hamner 1988; Johnston et al. 2005; Johnston and Read 2007). Such features have been shown to influence the availability and movements of nutrients, retention of plankton and aggregation of fish, and potentially provide increased foraging opportunities for predators (for review, see Benjamins et al. 2015). Although these hydrographic features may appear highly complex, there is generally an underlying temporal pattern to their structure. For example, water movements due to tidal processes are predictable in their timing, direction and velocity, and narrow channels can create temporally predictable water current patterns. For marine predators that must find prey that is often widely distributed in patches with complex spatial and temporal distributions, the underlying temporal predictability in potential foraging opportunities in marine habitat corridors may be important drivers in their use. Therefore, measuring how marine predators utilise narrow coastal channels, in combination with how this use is influenced by the underlying tidal pattern in corridor structure, is key to understanding the importance of habitat corridors in their foraging ecology.

In this study, we investigate in high resolution how a marine predator uses a dynamic habitat corridor. Specifically, we measure the spatial and temporal patterns of distribution and foraging effort exhibited by individual harbour seals around a narrow coastal channel subject to strong, tidally induced, water currents. This is carried out using data from animal-borne telemetry devices which record GPS locations and dive depth information, and shore-based observations, to which we apply a series of spatial analyses to quantify distribution in the channel. Furthermore, to understand the influence of variations in water currents on seal behaviour, we examine how these patterns vary with tidal state.

## Methods

### Harbour seals

Harbour seals are a relatively small species of Phocid seal averaging 1.5 m in length and around 70–100 kg in mass (Bjorge et al. 2010). They are distributed widely in coastal waters of the North Atlantic and North Pacific, where they use a variety of habitats to haul-out and breed, moult or rest (Thompson et al. 1997; Bjorge et al. 2010). In the UK, their annual life cycle comprises of aquatic mating (courtship and oestrous) in July, gestation, parturition and lactation in June–July and moult in August–September (Thompson and Rothery 1987; Thompson et al. 1994). Around the west and north of Scotland, they make foraging trips to sea that are generally within 25 km of their haul-out sites for periods of 1–2 days (Sharples et al. 2012). Prey most commonly identified around Scotland include sandeels (*Ammodytidae*), gadoids (whiting *Merlangius merlangus* and Atlantic cod *Gadus morhua*), flatfish (dab *Limanda limanda*, plaice *Pleuronectes platessa* and flounder *Platichthys flesus*) and in some regions salmonids (Atlantic salmon *Salmo salar* and sea trout *Salmo trutta*) (Thompson et al. 1996; Tollit and Thompson 1996; Pierce and Santos 2003).

### Study area

**Fig. 1:**
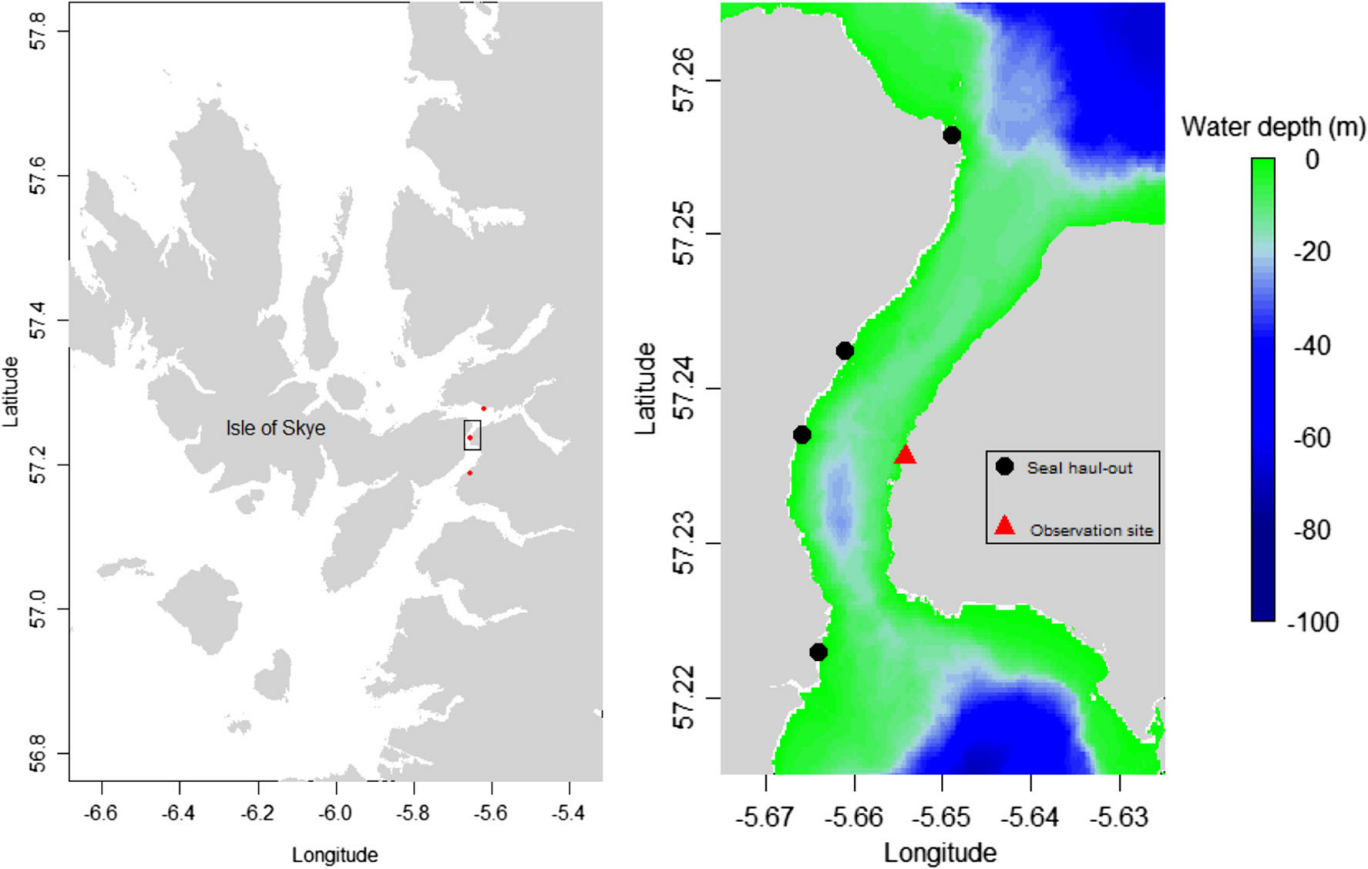
Map of the study area showing the location of the narrow tidal channel (Kyle Rhea) between the Isle of Skye and the mainland of Scotland. The locations of the 3 UHF receiving stations are also shown. The *rectangle* on the map denotes the area classified as the tidal channel in further analyses; the depth profile of the channel, together with the locations of the observation site and the seal haul-outs (identified using a combination of visual observations from shore and the location data from the animal-borne GPS tags), is shown in the *right panel*

The movements of individual harbour seals were studied in a narrow, tidally energetic channel on the west coast of Scotland (Kyle Rhea: 57° 14′ 8.10″ N, 5° 39′ 15.25″ W; Fig. 1). The channel runs from north to south, is approximately 4 km long and is 450 m wide at its narrowest point and 750 m at its widest. Water depths within the channel are generally less than 40 m (Fig. 1). Tidal currents within the channel can exceed 4 m s^−1^ at peak flow (Wilson et al. 2013) with water moving in a general northerly direction during the flood tide and a southerly direction during the ebb. The mean spring tidal height range is 4.5 m, and the mean neap range is 1.8 m (SeaGeneration (Kyle Rhea) Ltd 2012). During summer months (April–Sept), up to 85 harbour seals have been reported to haul-out on intertidal rocks along the sides of the channel (Cunningham et al. 2010) and are present in lower numbers (∼5 individuals) during other times of the year (SeaGeneration (Kyle Rhea) Ltd 2012).

### Telemetry

To measure the at-sea movements of harbour seals in the tidal channel, we deployed animal-borne GPS tags on 19 adult seals in April 2012 (three female and six male seals) and 2013 (five female and five male seals). Seals were captured within the channel whilst hauled out on intertidal rocks or in the water close to haul-out sites (Fig. 1) and anesthetised with Zoletil® or Ketaset® in combination with Hypnovel®. Capture and handling procedures are described in more detail by Sharples et al. (2012). The tags were attached to the fur at the back of the neck using Loctite® 422 Instant Adhesive.

In 2012, we deployed GPS/GSM tags (McConell et al. 2010) on seals; these are small (370 g: approximately 0.4 % of the average seal’s mass in air) data loggers that attempt to record the location of a seal at the surface at regular intervals (approximately every 15 min). Tags captured GPS data which were processed on board using the Fastloc® algorithm (Hazel 2009). Stored location and behavioural data were opportunistically relayed ashore by means of an embedded mobile phone (GSM) modem when the tag is within mobile phone coverage. The data were cleaned and erroneous locations removed using thresholds of residual error and number of satellites; tests on land using these thresholds showed 95 % of the cleaned locations had an error of <50 m (Russell et al. 2011). A pressure sensor on the tag provided seal depths at nine time intervals distributed equally in time throughout each dive.

In 2013, a different telemetry system was used; this combined data storage on animal-borne tags with periodic UHF transmission to archival base stations on shore. These tags did not record dive depth but the temporal resolution of movement tracks was higher than the 2012 deployment with locations recorded approximately every surfacing (approximately every 4 min). As above, the tags captured GPS data which were processed on board using the Fastloc algorithm (Hazel 2009). These semi-processed GPS data were stored on the tag to be downloaded via UHF to fixed base stations when animals had hauled out within range of a station for 30 min or more.

Three data archiving UHF base stations were placed at vantage points that overlooked nearby haul-out sites (Fig. 1). These were fully autonomous and powered by internal batteries charged by solar panels, and data were downloaded from the base stations periodically. As described above, these data were cleaned and erroneous locations removed using thresholds of residual error and number of GPS satellites. As these data were from animal-borne tags, they were considered to be blind and not subject to observer bias.

### Shore-based observations

Visual observations of seals in the channel were carried out from a cliff top overlooking the channel between the 3rd of June and 27th of July 2013. Scans were carried out at all states of the tide and during daylight hours between 0620 and 2130. Visual scans for seals at the water surface were made using binoculars (Monk Nereus 7 × 50) every 10 min, with scans lasting approximately 5 min in duration. To ensure that sampling was relatively consistent throughout the channel, scans were made at a steady speed and only in one direction (left–right and right–left; on alternate scans). The number of seals sighted in the water during each 10 min scan was noted. In addition, the number of seals hauled-out on intertidal rocks in the channel was counted every hour. A team of six observers collected data during the study; however, only a single observer collected data within an individual scan. Although the observations could not be blind, this part of the study involved describing temporal patterns in seal abundance and so did not involve hypothesis testing. Together with the multiple observer approach, this meant any potential bias as a result of individual preconceptions about tidal patterns in seal behaviour would be minimal.

### Statistical analysis: tidal patterns

Temporal patterns in the numbers of seals hauled out and in the water were modelled separately in General Additive Models (GAMs; Hastie and Tibshirani 1990). The abundance of animals in the channel was considered at two temporal scales: tidal state (flood-ebb cycle) and time of year. Tidal information (time in minutes from high tide) for each visual scan was extracted from the tidal prediction software POLTIPS (Version 3.4.0.3/10); tidal time and height information for Kyle Of Lochalsh (57.280 ° N, −5.705 ° W: ∼5 km from the channel) was used to determine the tidal state in the channel. There may also have been differences between observers in the probability of animals present being counted. Thus the explanatory covariates were a cyclic smooth of tidal state, a smooth of time of year (Julian day) and observer ID as a factor. The data comprised of observations collected close together in time, and consecutive observations are likely to be correlated beyond the underlying processes included in the model, resulting in some residual auto-correlation which violates a key assumption of GAMs. For this reason, GAMs with Poisson errors and a log-link function were fitted within a generalised estimating equation (GEE) framework to account for any residual auto-correlation. Typically, such data are seen as a collection of panels (we used day) within which model errors are permitted to be correlated and between which the errors are assumed independent. By using robust sandwich-based estimates of variance (Pirotta et al.. 2011), the uncertainty about the parameter estimates returned were robust to the presence of autocorrelation within each panel whilst not explicitly modelling this correlation.

### Statistical analyses: spatial distribution

To investigate the distribution of seals within the tidal channel, and how this varies with tidal state, we carried out a series of spatial analyses with data from the 19 tagged seals (during 2012 and 2013). The tag data consisted of a series of time-stamped GPS locations when the seal was at the water surface. Due to inherent variability in individual seal dive behaviour, there were potential spatial and temporal biases in the numbers of locations for each seal. To ensure that these did not affect the spatial analyses, the temporal period between cleaned locations (Russell et al. 2011) was standardised to a period of 10 min through linear interpolation between each reliable location.

The proportion of time in the water (interpolated at-sea GPS locations) that each seal spent within the channel (Fig. 1) was analysed using a generalised linear model with binomial errors and a logit-link function; the predictor variable was a factor describing the individual seal ID and response variable was the proportion of time each individual spent within the channel. The spatial distribution of each of the seals within the channel was analysed using a series of kernel estimates based on the geographical locations of the seals. All interpolated GPS data (i.e. including periods when the seal was hauled out) were used to estimate the spatial distribution of each individual. A fixed kernel density estimate was calculated using Equation 1 (Worton 1989); this was carried out for each seal individually and for the pooled data across all the seals.

1 $$ {\overline{f}}_h(x)=\frac{1}{n}{\displaystyle \sum_{i=1}^n}\frac{1}{h^2}K\left(\frac{x-{X}_i}{h}\right) $$

Where *K* is the uni-modal symmetrical bivariate probability density for a given grid point *x*.

*h*: is the smoothing parameter.

*X*: is a random sample of *n* independent points from an unknown utilisation distribution.

The spatial distribution of use was then plotted for each seal as a surface plot of the kernel densities for all grid points; as above, this was carried out for each seal individually and for the pooled data across all the seals. To ensure that the pooled estimate was not biased towards individuals that spent more time within the channel, the kernel densities for each individual were normalised to 1 and then summed to produce the pooled kernel density. In addition, changes in the distribution of seals in relation to the state of the tide were presented as a series of surface plots of the spatial distribution of seals within the channel using the same approach. Tide time and height information were used to divide the flood (when the current flows northwards through the channel) and ebb (when the current flows southwards) tides into three equal time periods in each (Ebb-start, Ebb-mid, Ebb-end, Flood-start, Flood-mid, Flood-end) and surface plots for pooled data across all the seals were created for each period.

### Dive behaviour

To assess the use of the water column within the channel, the diving behaviour of the GPS/GSM tagged seals (*n* = 9) was analysed; this was carried out to provide a measure of foraging effort by the seals. The best estimate of the swimming track of a seal was taken to be the straight line between successive GPS locations. Given that the tags attempted to record GPS locations every 8 min, a proportion of the start or end points of the dive did not have associated GPS locations. The possible error in the location estimate will increase with time from the nearest location fix. In order to minimise potential location error, we only used dive information where either the start or end of the dive was a recorded GPS location fix. Based on these locations, the location and depth of each seal was estimated at 10 s intervals during dives by interpolating the XY position assuming direct straight line movement between the start and end of each dive and linearly interpolating between successive time depth records; further, the maximum depth of each dive in relation to the depth of the seabed at the corresponding location was calculated by adding the tidal height (derived from POLTIPS, National Oceanographic Centre, NERC) to the measured maximum dive depth from the pressure sensors on the tags. The start of each dive was defined as being when the tag was wet and deeper than 1.5 m for a period of 8 s and the end was defined as being when the tag was dry or shallower than 1.5 m. For the flood and ebb tides, dive parameters including the dive duration (secs), the post dive surface interval (secs), and the proportion of dives to the seabed (defined as a dive in which the maximum depth was greater than 95 % of the depth of the seabed at that location) were summarised for each individual during periods when they were within the channel (Fig. 1).

## Results

### Tidal patterns

Seals were sighted in the water during 1332 (96 %) of the shore-based scans; the mean number of seals sighted in the water was 6.9 (SD = 6.1) and ranged from 0 to 39. Seals were sighted hauled out on shore during 196 (93 %) of the haul-out scans; the mean number of seals hauled out each hour was 33.2 (SD = 29.3) and ranged from 0 to 112.

**Fig. 2:**
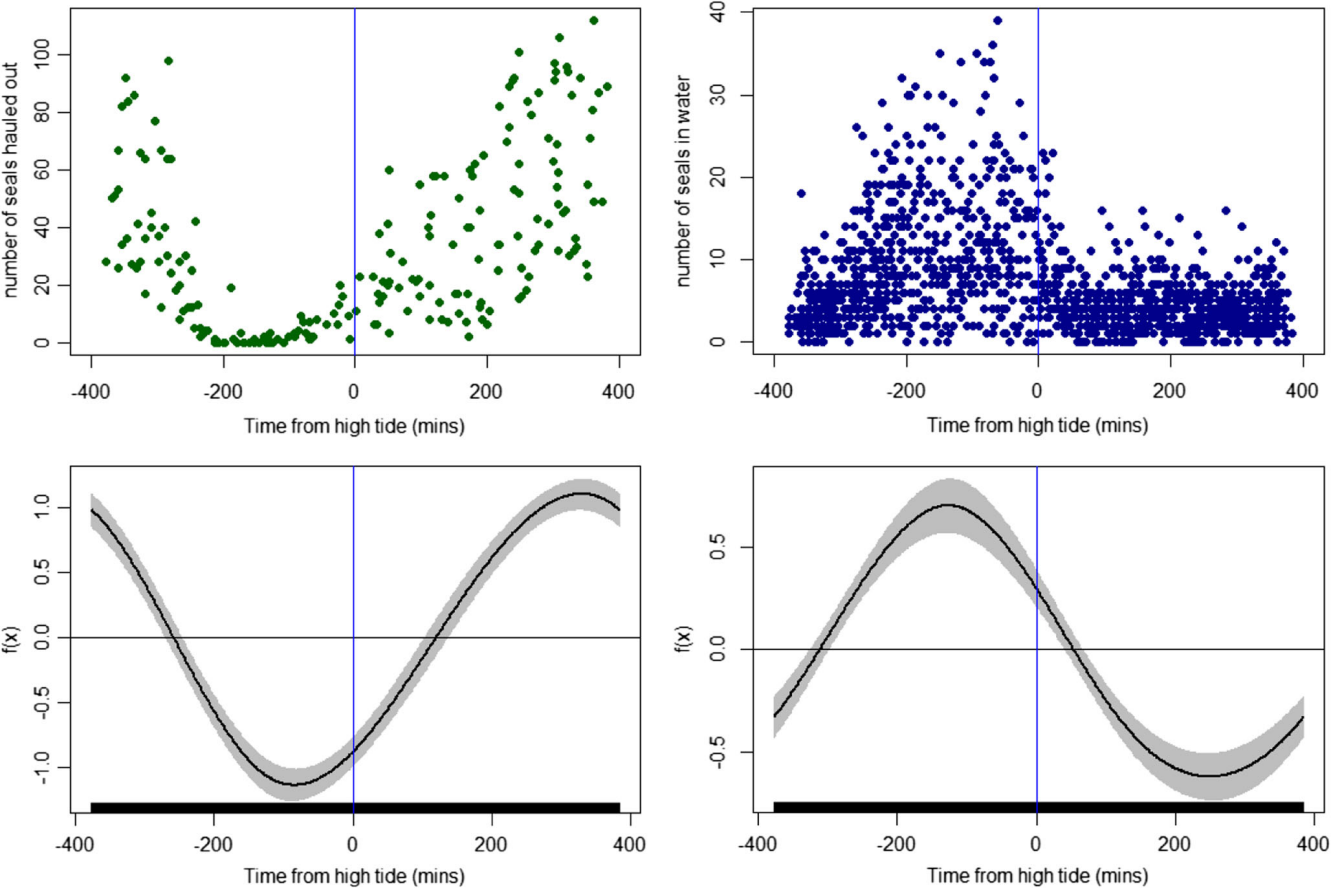
Temporal pattern in the numbers of harbour seals sighted in the narrow tidal channel in relation to the state of the tide. The figure shows counts made from shore of seals hauled out in the channel (*top left*) or sighted in the water (*top right*), with associated generalised additive model functions (on the link scale) of seal numbers in relation to tide shown below each plot

Using Akaike’s Information Criterion (AIC) for model selection, we found that observer ID, Julian day, and tidal state were all predictors of both the numbers of seals sighted in the water and the numbers of seals hauled out on shore. Numbers of seals sighted in the water were markedly higher during the flood tide compared to the ebb tide and this was reflected in the GAM model functions; the predicted numbers of seals in the water were at a minimum around 1–2 h before low tide but rapidly increased during the flood tide to peak around 1–2 h before high tide (Fig. 2). In contrast, numbers of seals sighted hauled out were markedly lower during the flood tide compared to the ebb tide with numbers hauled out predicted to peak around 1–2 h before low tide and were at a minimum around 1–2 h before high tide (Fig. 2).

### Spatial distribution

**Fig. 3:**
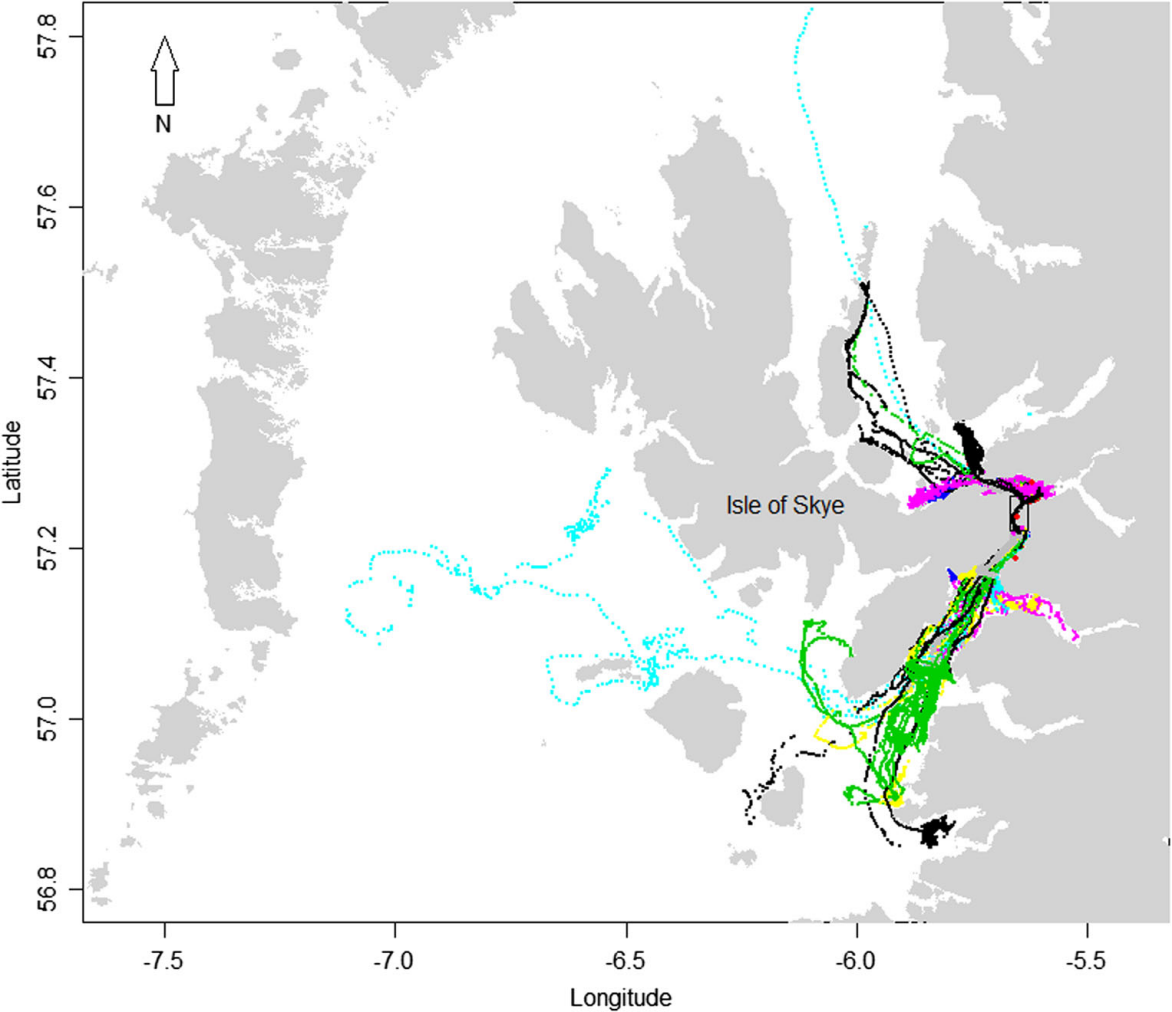
Map of the study area showing the location of the narrow tidal channel (denoted by the *rectangle*) between the Isle of Skye and the mainland of Scotland and the telemetry tracks from harbour seals tagged within the study area during 2012 (*n* = 9) and 2013 (*n* = 10). The locations are colour coded to represent the track of a different individual

The duration over which each of the 19 GPS tags collected location data varied from 15 to 99 (mean = 57; SD = 28) days in 2012 and from 40 to 98 (mean = 72; SD = 18) days in 2013. During the study, all seals made a number of trips outside the tidal channel travelling several tens of kilometres from the channel; the furthest distance travelled from the channel was around 200 km (Fig. 3). The proportion of time at sea that individuals spent within the channel varied from 0.19 to 0.72 (mean = 0.51; SD = 0.17) (Table 1). There were no significant differences in the proportion of time spent at sea in the channel by each sex ($GLM:{\upchi}_{18}^2$ =0.01, *P* = 0.91) or between the 2 years ($GLM:{\upchi}_{18}^2$ = 0.18, *P* = 0.67). The proportion of the study period seals were hauled out within the channel varied from 0.04 to 0.30 (mean = 0.15; SD = 0.07; Table 1); altogether, seals spent 0.25 to 0.97 (mean = 0.66; SD = 0.20) of the study within the channel (haul-out and at-sea combined). Further, when within the channel, there was inter-individual variation in the proportion of their time spent hauled out (when expressed as a proportion of their total time within the channel); this varied from 0.08 to 0.49 (mean = 0.23; SD = 0.09). Although there was clear variation in the use of the channel by individuals, the majority (15/19) spent more than 0.5 of the study period within the channel and seven spent more than 0.75 of the study period in the channel.

**Table 1:** Summary of the tagged seals in the study including the year of deployment, sex, mass (kg), the duration of tag deployment (days) and the proportion of total time spent in the water within the channel or hauled out within the channel

Tag ID	Year	Sex	Mass (kg)	Tag duration (days)	Proportion of time in channel	
					In water	Hauled out
64001	2012	Male	72.0	78	0.62	0.19
64002	2012	Male	70.4	43	0.61	0.24
64003	2012	Male	87.0	93	0.19	0.18
64004	2012	Male	77.2	99	0.72	0.25
64005*	2012	Female	74.6	46	0.65	0.15
64006	2012	Female	83.0	49	0.34	0.14
64007	2012	Male	92.0	15	0.56	0.05
64008	2012	Female	83.4	57	0.22	0.04
64009	2012	Male	79.6	31	0.19	0.06
65154	2013	Female	82.6	75	0.67	0.18
65155	2013	Female	76.2	67	0.62	0.19
65156	2013	Male	81.6	54	0.46	0.13
65157	2013	Male	89.4	96	0.48	0.16
65159	2013	Male	80.2	98	0.44	0.09
65161	2013	Female	86.4	59	0.61	0.10
65162	2013	Male	68.2	67	0.58	0.13
65163	2013	Male	87.2	82	0.61	0.18
65164	2013	Female	76.0	80	0.63	0.10
65165*	2013	Female	78.4	40	0.44	0.30

All tags were deployed in April of the respective year. It should be noted that a female seal tagged in 2012 was recaptured in 2013 (denoted by an asterisk)

**Fig. 4:**
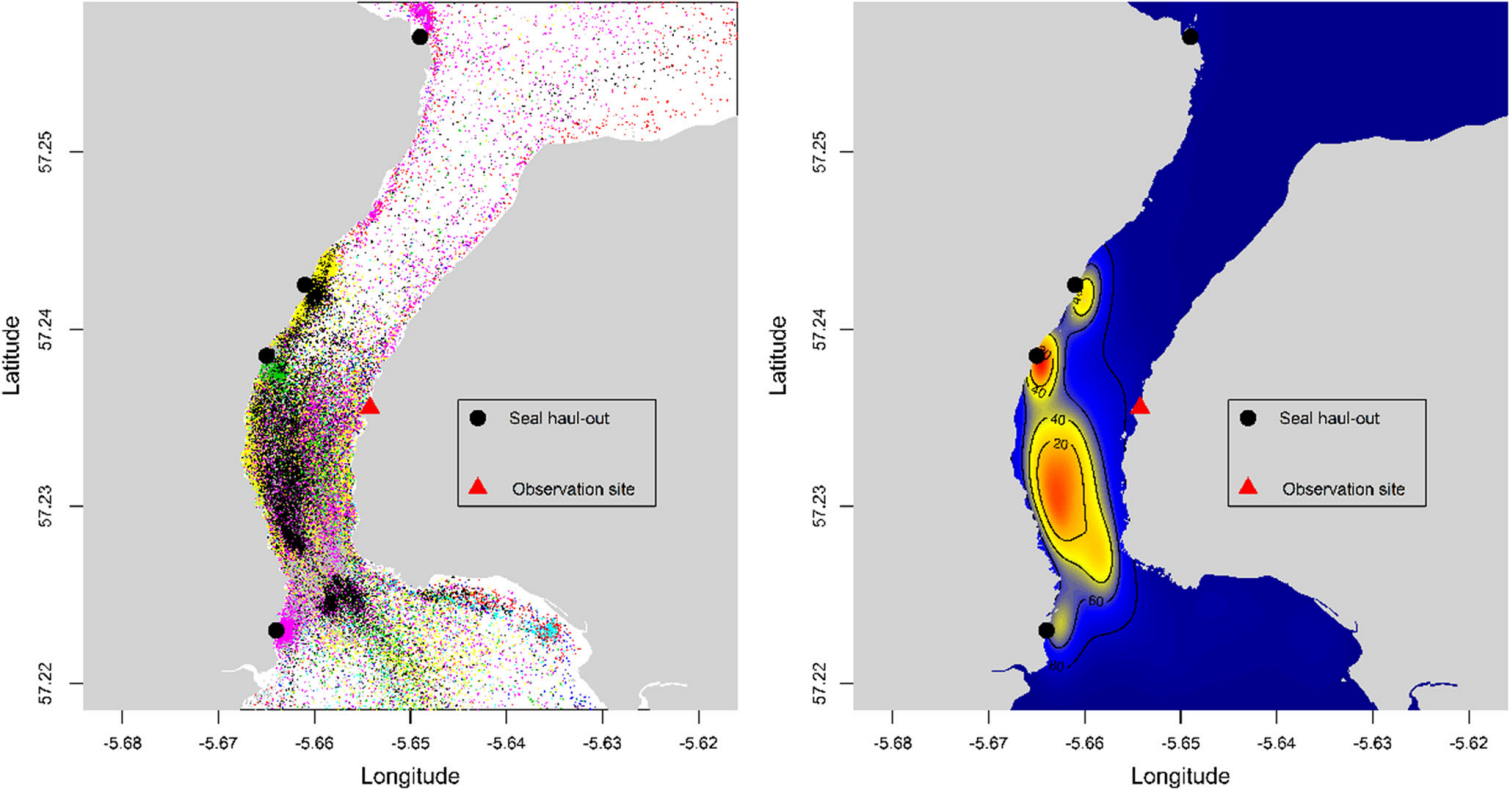
Spatial distribution of harbour seals in the narrow tidal channel. The *left panel* shows the locations of tagged seals within the channel (colour coded by individual) and the *right panel* shows the surface plots of the spatial distribution colour coded for relative frequency of occurrence (low = *blue*, high = *red*) for the pooled data across all the seals

The surface plot of the kernel densities for the pooled location data for all seals revealed the existence of a highly localised hot-spot of at-sea usage around the southern end of the tidal channel close to its narrowest point. In addition, the locations of the haul-out sites at the western side of the channel were evident in the surface plot. In contrast, the use of the northern end of the channel by the seals was relatively low (Fig. 4, Supplemental material: Fig. S1).

**Fig. 5:**
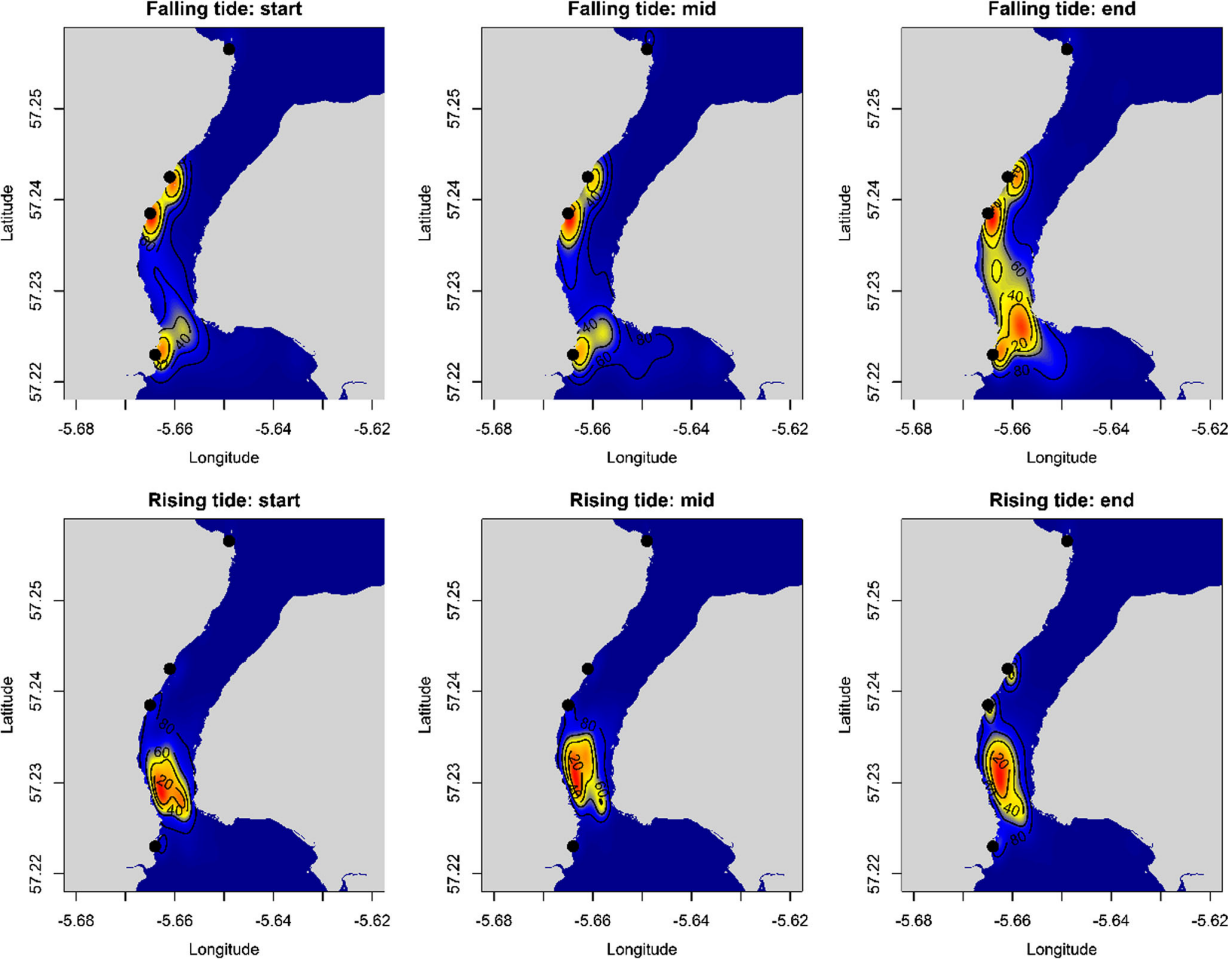
Tidal pattern in the spatial distribution of harbour seals in the narrow tidal channel. The figure shows a series of surface plots of the spatial distribution colour coded for relative frequency of occurrence (low = *blue*, high = *red*). Tide time and height information were used to divide the flood (water flows from south to north) and ebb (water flows from north to south) tides into three equal time periods in each (Ebb-start, Ebb-mid, Ebb-end, Flood-start, Flood-mid, Flood-end) and surface plots for pooled data across all the seals

The surface plots of kernel densities for the pooled data by tidal state revealed a distinctive tidal pattern in the use of the channel (Fig. 5). Throughout the ebb tide, the areas of highest usage were located on and around the haul-out areas on the western side of the channel. At sea usage of the channel was remained relatively low until the period 0–2 h before low tide, when seals left the haul-out areas and moved to the southern part of the channel. The discrete hot-spot at the southern end of the channel became apparent after low tide and continued throughout the flood tide (Fig. 5). Throughout all periods, the use of the northern part of the channel remained low.

Analysis of the diving behaviour within the channel showed that all seals for which we had dive data (9 individuals; 21,974 dives) carried out dives in the channel with median dive durations (±SD) for each individual ranging from 60.0 (72.3) to 192.0 (131.9) seconds and median post-dive surface durations ranging from 32 (107.9) to 68 (351.5) seconds. The proportion of dives close to the seabed varied between 0.50 and 1.00 for each individual (Table 2). When compared between the flood and ebb tides, the majority of seals (7/9) had longer median dive durations during the flood tide than the ebb tide, the majority (5/9) had shorter median post-dive surface durations, all had lower median surface duration: dive duration ratios during the flood than ebb tide, and the majority (6/9) had a higher proportion of dives to the seabed during the flood than ebb tide (Table 2).

**Table 2:** Summary of the dive parameters for each seal when diving in the tidal channel

Tag ID	Dive duration (secs)		Surface duration (secs)		Surface duration: dive duration ratio		Prop. of dives to seabed	
	Flood tide	Ebb tide	Flood tide	Ebb tide	Flood tide	Ebb tide	Flood tide	Ebb tide
64001	152.0 (66.6)	140.0 (82.7)	40.0 (66.5)	52.0 (323.0)	0.25 (3.09)	0.34 (4.63)	0.58	0.50
64002	148.0 (101.8)	44.0 (78.8)	36.0 (56.4)	20.0 (189.7)	0.20 (1.49)	0.25 (5.34)	0.82	0.77
64003	104.0 (76.3)	52.0 (86.5)	40.0 (245.0)	48.0 (519.1)	0.36 (8.73)	0.65 (20.15)	0.70	0.65
64004	112.0 (75.9)	32.0 (49.1)	36.0 (90.2)	28.0 (176.8)	0.29 (5.56)	0.60 (8.92)	0.75	0.74
64005	180.0 (76.6)	184.0 (83.0)	44.0 (37.9)	48.0 (86.5)	0.23 (1.31)	0.25 (2.19)	0.60	0.74
64006	128.0 (82.7)	52.0 (71.1)	44.0 (93.4)	44.0 (386.2)	0.32 (6.08)	0.52 (4.50)	0.74	0.75
64007	188.0 (134.4)	208.0 (129.3)	64.0 (315.8)	76.0 (82.6)	0.27 (6.17)	0.32 (15.54)	0.73	1.0
64008	144.0 (87.3)	112.0 (86.8)	48.0 (129.5)	44.0 (782.3)	0.32 (4.38)	0.35 (3.96)	0.80	0.74
64009	152.0 (72.6)	124.0 (69.9)	36.0 (88.1)	40.0 (66.5)	0.23 (4.32)	0.33 (34.17)	0.71	0.58

Dive data were collected from 9 seals during 2012. The table shows the median values (with standard deviation in parentheses) for dive duration, post dive surface duration, the post dive surface duration to dive duration ratio and the proportion of dives where the maximum depth was close to the seabed (within the lower 5 % of the water column) for the flood and ebb tides. The majority of seals (7/9) had longer median dive durations during the flood tide than the ebb tide, the majority (5/9) had shorter median post-dive surface durations, all had lower median surface duration: dive duration ratios during the flood than ebb tide, and the majority (6/9) had a higher proportion of dives to the seabed during the flood than ebb tide

## Discussion

This paper presents the results of a study which combined both individual and population data to demonstrate the behaviour of a mobile marine predator in a narrow, coastal channel subject to strong tidal currents. Results show that, during the study, individual harbour seals spent a significant proportion of their time in the narrow channel diving throughout the water column; although all tagged seals did carry out trips outside the channel, 15 (79 %) of them spent the majority (over 50 %) of their time within the confines of the channel. Furthermore, analysis of their spatial distribution within the channel revealed a striking pattern with the majority of the tagged seals spending a high proportion of their time close to its narrowest point (Supplemental material: Fig. S1).

In general, harbour seals forage at ranges of several tens of kilometres from their haul-out sites whilst exhibiting high inter-individual variation in their distribution at sea (Sharples et al. 2012). For example, harbour seals tagged elsewhere in west Scotland travelled a mean of 10.5 km from haul-out sites with some seals travelling more than 100 km (Cunningham et al. 2009); mean distances from haul-outs in other regions of the UK ranged from 10.8 to 152.6 km (Sharples et al. 2012). Whilst at sea, a number of studies have shown high site fidelity by individual seals with individuals selecting relatively discrete foraging areas at sea (Suryan and Harvey 1998; Tollit et al. 1998; Thomas et al. 2011; Peterson et al. 2012). However, there is often a high degree of inter-individual variability in foraging site fidelity, with individuals exhibiting disjunct spatial use at sea (Bjorge et al. 1995; Peterson et al. 2012). Although, there are previous studies where multiple tagged harbour seals appear to show a relatively high degree of overlap in their at-sea usage (Suryan and Harvey 1998; Peterson et al. 2012), this has generally been at a scale of ∼25 km^2^. The results of the current study with 15 of the tagged seals (79 %) spending the majority (over 50 %) of their time within the confines of the small part of a narrow channel (∼0.5 km^2^) appears relatively unusual.

Overall, relatively high numbers of seals were sighted in the channel; the mean number of seals sighted in the water during 5 min scans was 6.9 (max = 39) and the mean number sighted hauled out on shore was 33.2 (max = 112). An important consideration when interpreting these count data is that, when in the water, pinnipeds generally spend a significant proportion of their time submerged and are therefore unavailable to be counted at the surface; evidence shows that grey seals (*Halichoerus grypus*) can spend approximately 89 % of the time submerged (Thompson and Fedak 1993). The marked difference between haul-out and in-water counts is therefore likely to be a direct consequence of this proportion of seals in the water being submerged at the time of visual scans.

There was also a distinctive tidal pattern in the use of the narrow channel by the seals. Sightings in the water peaked during the flood tide (when the current flowed northwards) around 1–2 h before high tide and were at a minimum during the ebb (southward) tide around 1–2 h before low tide; conversely, numbers of seals that were hauled out peaked during the ebb tide and were at a minimum during the flood tide. These results support preliminary surveys in this area which observed that hauled-out seal numbers increased during the ebbing tide, with highest numbers observed from about 3.5 h before low tide until half an hour after (Cunningham et al. 2010). So in addition to the in-water behaviour, the haul-out patterns appear relatively unusual and contrasts with the more traditional understanding that haul-out behaviour peaks at the transition of ebb and flood tides (Schneider and Payne 1983; Pauli and Terhune 1987); this adds to increasing evidence that haul-out patterns of harbour seals in some locations may not be driven by tide but by a complex interaction of environmental variables such as diel rhythms (Calambokidis et al. 1987; Hamilton et al. 2014), predation risk (London et al. 2012) and weather conditions (Schneider and Payne 1983; Grellier et al. 1996).

When studying how seals behave in relation to tidal state, an important caveat needs to be considered. Specifically, given that the availability of haul-out sites is often restricted to particular tidal states [usually during low tide, (Pauli and Terhune 1987)], it is important to avoid the conflation of at-sea patterns that are driven by tidal currents with those that are driven by haul-out site availability. In areas where haul-out availability appears to be the primary driver behind haul-out behaviour, numbers of seals counted at equal times either side of low tide (when presumably the same area of haul-out space is available) are generally similar. For example, Thompson et al. (2005) analysed haul-out counts of seals from the Moray Firth, Scotland with respect to tidal state; counts made 2 h before low tide were similar (within ∼10 %) of counts made 2 h after low tide. In the current study, haul-outs were evidently available to seals throughout the tidal cycle (Fig. 2), and the marked difference in haul-out numbers 2 h either side of low tide suggest that the pattern observed was not primarily driven by haul-out site availability but by the temporal patterns in the water currents in the adjacent channel (Fig. 2).

Further caveats associated with surveys in dynamic environments need to be considered. Specifically, due to the nature of the surveys, it is likely that a larger area of water was surveyed during a fixed period of time when the tide is flowing in comparison to when it is slack. Within a 5-min scan of the study area, a shore-based observer would effectively survey an area of approximately 2.6 km^2^ of water during periods of peak tidal flow (4 ms^−1^), as opposed to an area of approximately 2 km^2^ during no flow. This potential bias could therefore have manifested itself as a perceived 30 % increase in the number of seals during both the flood and ebb tides. However, the marked disparity in the number of seals sighted in the water between peak flood tides and peak ebb tides (when the tidal flow speeds are likely to be relatively similar) is far greater (∼200 %) than could be explained by a 30 % increase in survey coverage (Fig. 2). This, together with the patterns of usage observed from the telemetry data, strongly suggests that the patterns observed in the land based observations are driven by real changes in seal behaviour rather than methodological biases.

It appears therefore that, at least during the summer months (April–August), the southern end of the narrow channel is targeted by these harbour seals. In other areas, distinctive spatial and temporal patterns in the use of such geographic constrictions by harbour seals have been linked to reproduction. Specifically, during the breeding season (June–July), male seals have been shown to use narrow channels to maximise encounters with females moving between haul-out locations and offshore foraging areas; the highest densities of male seals producing breeding vocalisations were found along narrow constrictions in the transit routes between haul-out sites and foraging areas (Van Parijs et al. 1999, 2000). In Scotland, display behaviour by male seals generally occurs during a relatively short period from the start of July where males make characteristic dives (a long surface interval of 20–30 s followed by a series of three to five short dives of around 60 s) (Van Parijs et al. 1997); however, both male and female seals exhibited the same localised spatial and temporal distributions throughout the current study and had similar duration dives so it seems unlikely that display behaviour is the primary driver behind the pattern. Further, although females generally restrict their foraging ranges during the pupping season, elsewhere in Scotland this appears to be a relatively short period (2–3 weeks) from around mid-June (Thompson et al. 1994). Females in our study used the channel throughout the study period and only a single pup was observed in the water or hauled out during the land based visual observations; it therefore seems unlikely that this area was used as a pupping location and that an associated range contraction by pupping females is unlikely to fully explain the highly localised spatial patterns.

Within the channel, all seals tagged with depth recorders made prolonged dives underwater with between 50 and 100 % of the dives being made close to the seabed; this together with the highly localised spatial distribution over periods of several months strongly suggests that the seals were foraging within the channel. Further, when compared between the flood and ebb tides, the majority of seals had longer median dive durations, shorter median post-dive surface durations, a higher proportion of dives to the seabed, and all had lower median surface duration: dive duration ratios during the flood than ebb tide. When viewed together, these results suggest higher foraging intensity during the flood than the ebb tide within the channel (Lesage et al. 1999; Baechler et al. 2002; Beck et al. 2003).

There is some evidence to suggest that seals may be attracted to such areas due to enhanced foraging opportunities or efficiency (Brown and Mate 1983; Thompson et al. 1991; Suryan and Harvey 1998; Zamon 2001). Brown and Mate (1983) reported harbour seals foraging on salmon during the incoming tide at a narrow constriction in Netarts Bay (Oregon, USA). In a tidal strait in San Juan Islands, Washington State, Zamon (2001) studied the temporal and spatial patterns of Pacific harbour seals in relation to tidal phase. Counts of seals at the water surface were made from shore and were compared between different states of the tide. Results support the patterns observed in the current study; seal sightings were highest near the most constricted part of the channel and that there was a clear tidal pattern in seal presence in the channel with greatest median counts during flood tides. In terms of foraging, Zamon (2001) showed that observations of large fish captures by seals peaked during the flood tide at the most constricted part of the channel. It was hypothesised that, rather than expending energy searching for prey in large, open volumes of water, harbour seals may choose to focus effort in a smaller volume where topography causes either encounter rates, prey density or vulnerability of prey to be greater than in surrounding habitat (Zamon 2001). However, without information on individual movements in this previous study (Zamon 2001), it was not possible to confirm whether the pattern of sightings represented individual seals with focused foraging effort at the constricted part of the channel as hypothesised or whether it simply represented the bottleneck effect of an increase in seal density as individuals moved through the narrow constriction.

To maximise their foraging efficiency, predators have been shown to make use of narrow constrictions in habitat (e.g. narrow valleys, forest firebreaks, drainage ditches) to increase prey encounter rates or prey capture efficiency by intercepting prey using these corridors for regular movement (e.g. Gard 1971; Hvidsten and Mokkelgjerd 1987; Hvidsten and Lund 1988; Brinkerhoff et al. 2005; Šálek et al. 2009; Knowlton and Graham 2010). Narrow coastal channels such as the one in the current study can be effectively viewed as hard-edged habitat corridors for mobile marine species [where individuals cannot cross to enter habitats at the sides of the corridor (Stamps et al. 1987)]. Our results confirm that, within the tidal channel studied here, individual seals do spend a significant proportion of their time within the channel and focus their foraging effort at the most constricted part of the channel. Although foraging success or prey densities were not quantified in the current study, frequent observations during the shore-based scans were made of seals at the surface with Atlantic mackerel (*Scomber scombrus*) in their mouths; this suggest that tidal patterns in the availability of this prey species may underpin the spatial and temporal pattern shown by the seals.

Atlantic mackerel is a pelagic schooling fish which, on the west coast of Scotland, appears in the diets of many marine predators including marine mammals (Pierce and Santos 2003; Santos et al. 2004) and seabirds (Nogales et al. 1995). They are a highly mobile species and make relatively long migratory movements in dense schools in coastal waters (e.g. Walsh et al. 1995). During summer, they make northwards migrations along the west of Scotland (Reid et al. 1997); this is supported by dietary evidence which shows mackerel in the diet of harbour seals between June and September but an absence between October and May (Pierce and Santos 2003). It is therefore plausible that the channel in our study acts as a coastal movement corridor for mackerel during the summer.

Unlike most terrestrial habitat corridors where the underlying habitat features remain relatively fixed, marine corridors such as narrow channels are in constant flux as a result of tidally driven water currents. In the current study, water movements within the channel can exceed 4 m s^−1^ at peak flow (Wilson et al. 2013); this is beyond the maximum sustainable swimming speed of most fish species (Blaxter and Dickson 1959) and it seems likely therefore that fish will also be actively forced through the channel during peak tidal currents. It is clear that mobile predators such as harbour seals looking to benefit from foraging here would also be subject to the same high flow speeds as their prey. To effectively forage, the seals would require a means of maintaining their position within the channel without incurring excessive energetic costs of swimming against the water current, bearing in mind that the energetic cost of swimming increases markedly at speeds greater than 1.5 m s^−1^ (Hind and Gurney 1997) and the maximum burst speed for harbour seals is around 4 m s^−1^ (Williams and Kooyman 1985). Potential mechanisms for this have been documented in many river dwelling fish species and include flow refuging (where the animal exploits regions of reduced flow relative to the channel) or vortex capturing (harnessing the energy of environmental vortices or eddies) (for review, see Liao 2007). There is also evidence of marine mammals exploiting dynamic systems through selection of areas with reduced velocity; for example, Johnston et al. (2005) showed that minke (*Balaenoptera acutorostrata*) and fin (*Balaenoptera physalus*) whales in a tidally-driven vortex focus their movements within slower velocity regions of the wake to exploit prey aggregations occurring there. However, the tactics used by the seals in the current study remain unknown at this time.

Seals may also be attracted to narrow channels, not in response to absolute prey abundance, but rather as a result of increased prey capture efficiency. Concentrating foraging efforts at times and locations of increased prey vulnerability has been shown to significantly enhance foraging success of a range of terrestrial and marine predators (Quinn and Cresswell 2004; Hopcraft et al. 2005; Crook and Davoren 2014). Narrow channels such as the one in the current study are tidally energetic; strong turbulence provides a potential mechanism to disorient prey and imposes a metabolic cost as prey try to maintain orientation (Zamon 2002; Liao 2007). Strong currents can also influence cohesion among schooling species (Gómez-Gutiérrez and Robinson 2006; Robinson et al. 2007), potentially leading to the breakup of schools which facilitates predation of individuals (Vabø and Nøttestad 1997; Enstipp et al. 2007); recent evidence shows that harbour seals foraging on schooling prey (herring: *Clupea harengus*) are more successful when small groups or a single fish are separated from the main school (Kilian et al. 2015).

Irrespective of whether increased prey abundance and/or availability enhance foraging success in narrow channels, an inherent temporal predictability in prey occurrence due to tidal currents may result in increased foraging efficiency for individual predators. For example, high density prey patches may be available dispersed throughout a surrounding coastal marine habitat (Irons 1998; Weimerskirch 2007); however, if the locations of these patches are relatively unpredictable in space and time, it may prove more efficient for a predator to focus search efforts in a lower prey density area but which has a predictable temporal persistence. This may be particularly true for central place foraging species with constrained at-sea time budgets (Orians and Pearson 1979).

In summary, the results of this individual-based tracking study combined with spatially focussed shore-based observations has shown that harbour seals exhibit intensive use of a highly localised part of a narrow coastal channel subject to strong tidal currents. In addition, all tagged seals showed a marked response to tidal state with each of them using the same discrete region of the channel during the flood tide. Although the underlying mechanism of this behaviour is uncertain, it seems likely that the spatial and temporal patterns are related to increased prey abundance or availability, or enhanced foraging efficiency. The results highlight the importance of this coastal channel as a dynamic habitat corridor for this marine predator. To reveal how the movements and foraging interactions between seals and their prey actually occur, mapping of the three dimensional water movements throughout the channel in combination with underwater observations of foraging individuals would be rewarding. This would help unlock whether prey abundance or an availability mechanism is driving this unusually distinctive seal behaviour pattern.

## Electronic supplementary material

Fig. S1 (DOCX 601 kb)
