# Supplementary material for: Dynamic habitat corridors for marine predators; intensive use of a coastal channel by harbour seals is modulated by tidal currents
Source: Behav Ecol Sociobiol. 2016 Oct 14;70(12):2161–74. doi: 10.1007/s00265-016-2219-7 (PMC5102963; doi:10.1007/s00265-016-2219-7)
Supplement: Supplementary file 1 — (DOCX 601 kb) [file 265_2016_2219_MOESM1_ESM.docx]

Electronic Supplemental Material.

Dynamic habitat corridors for marine predators; intensive use of a coastal channel by harbour seals is modulated by tidal currents

Behavioral Ecology and Sociobiology

Gordon D. Hastie 1,* Deborah J.F. Russell, Steven Benjamins, Simon Moss, Ben Wilson, & Dave Thompson

*corresponding author: gdh10@st-andrews.ac.uk


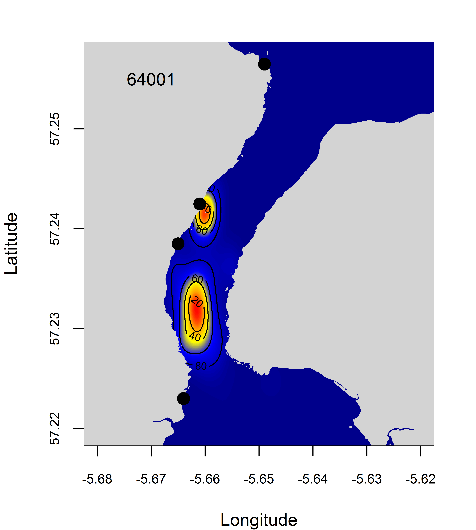

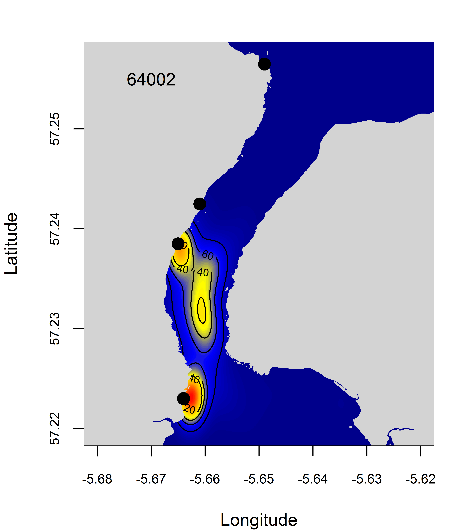

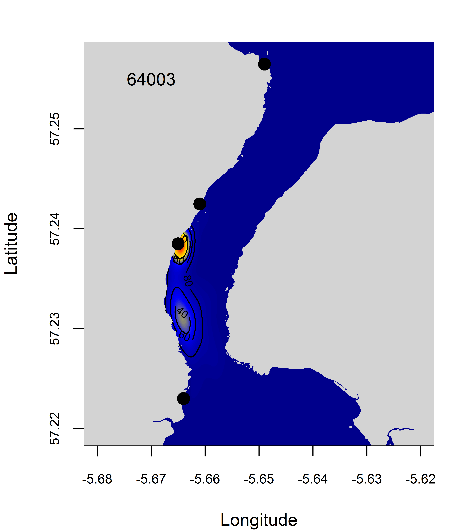

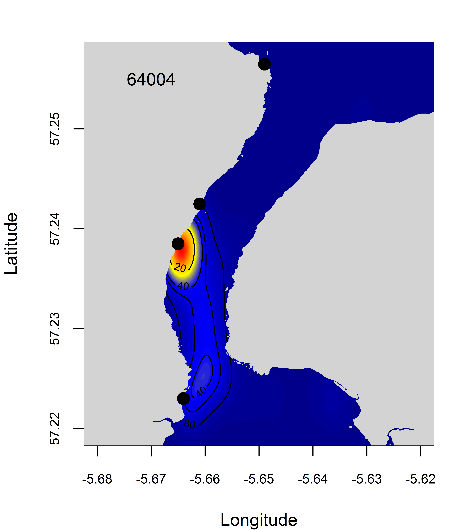

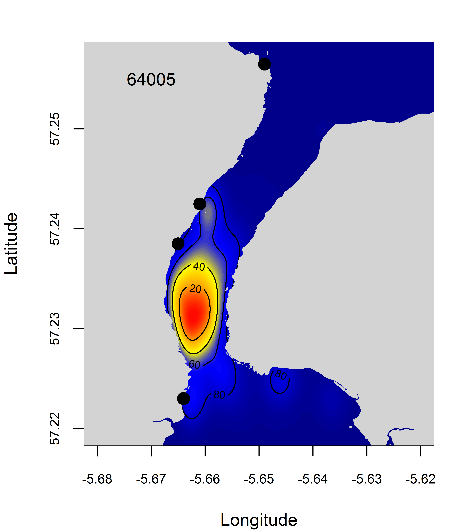

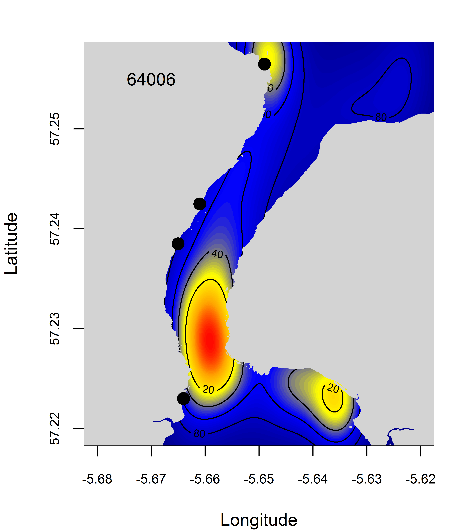


Figure S1: see below


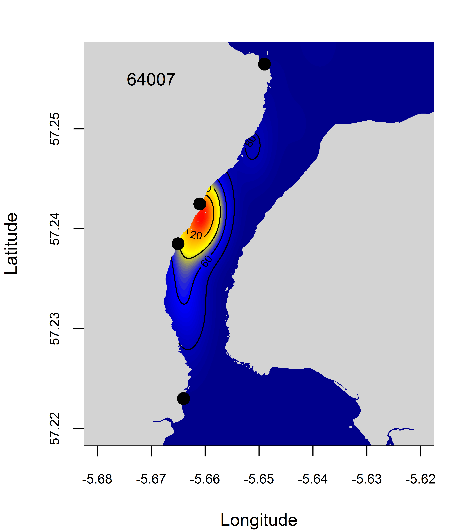

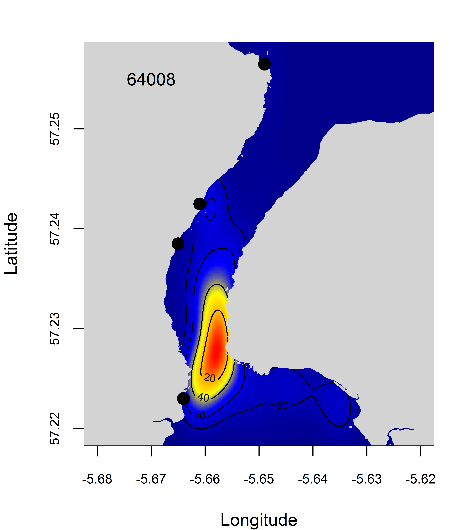

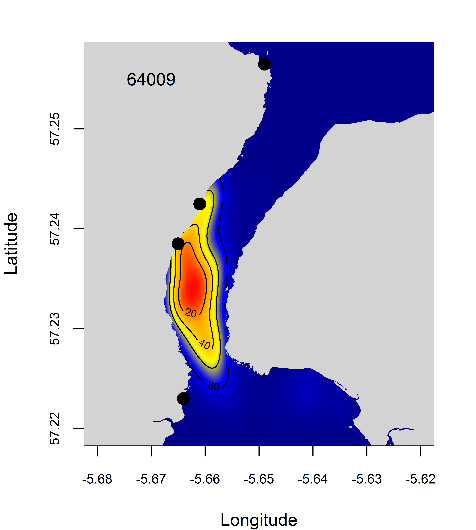

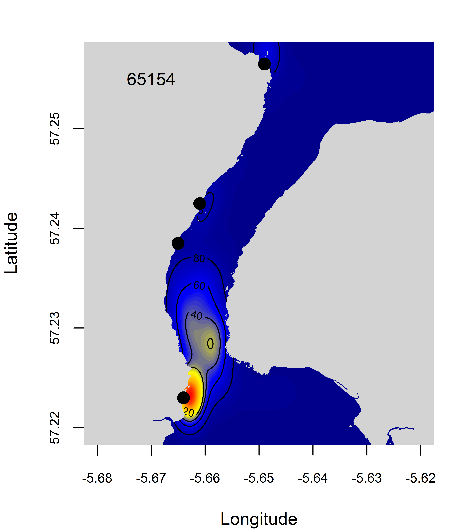

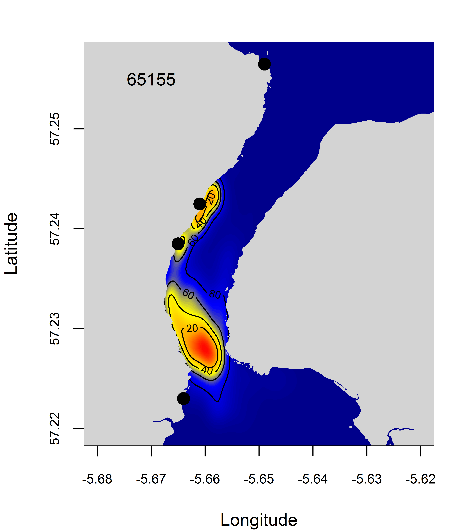

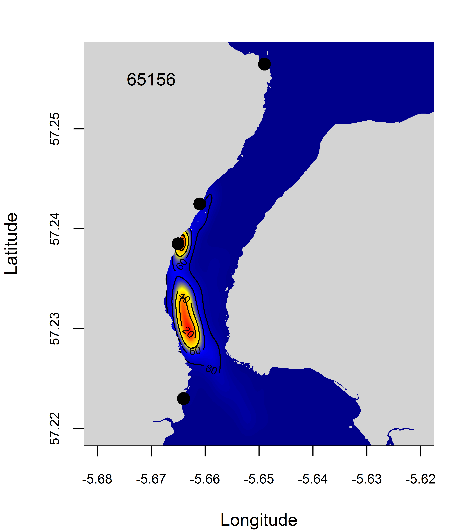


Figure S1: see below


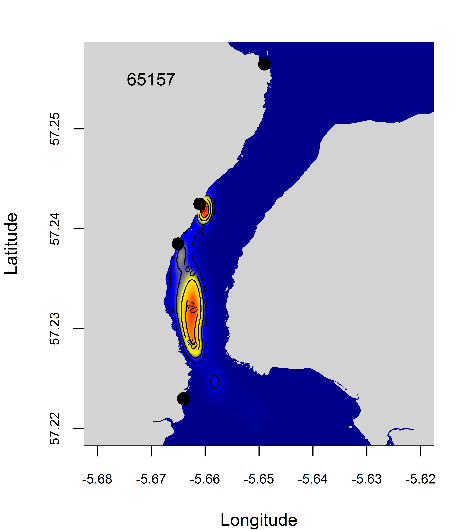

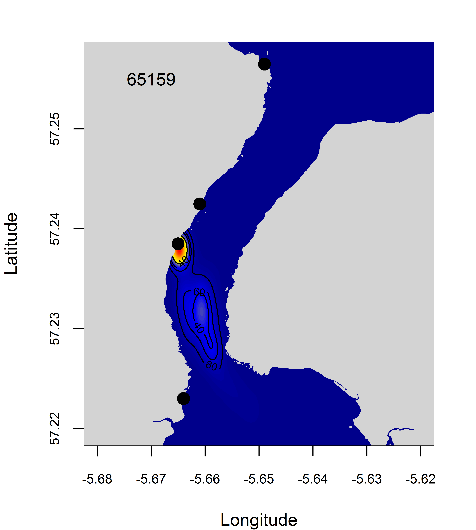

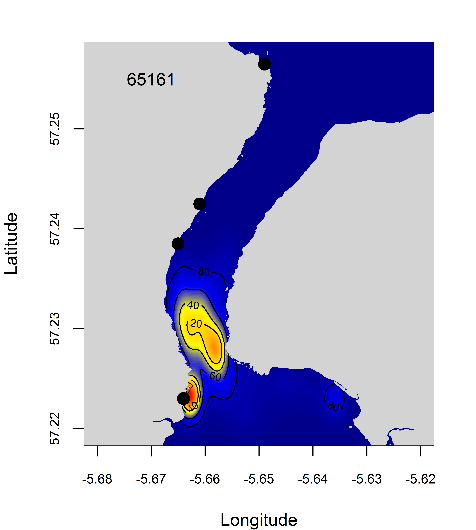

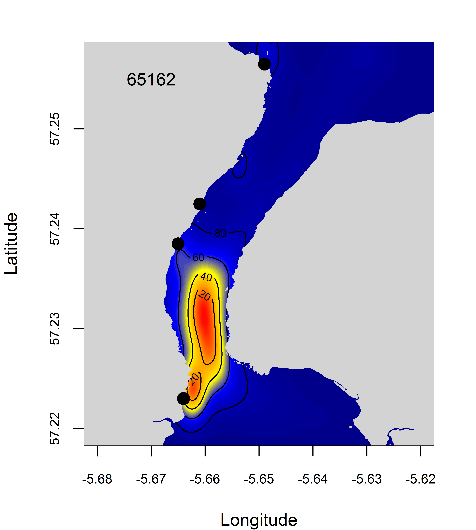

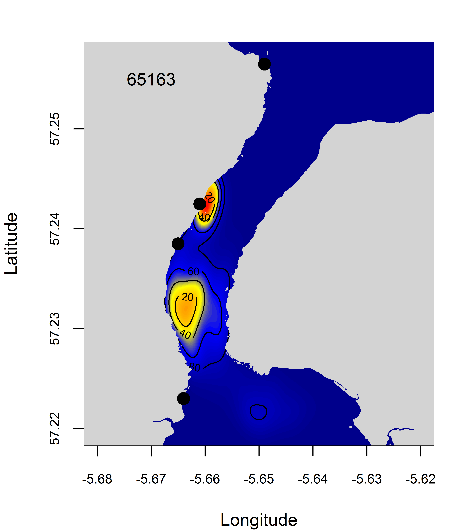

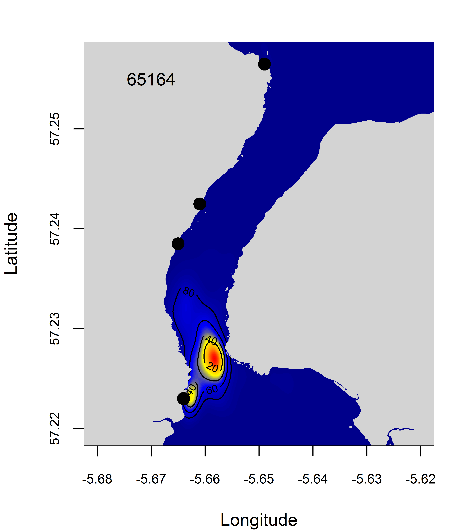

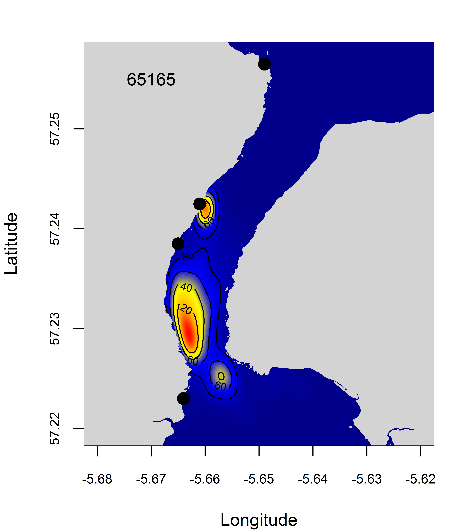


Figure S1: Plots of the spatial distributions of each individual harbour seal within the channel; each plot is a surface of the kernel densities for all grid points are shown where of the colour coding represents the relative frequency of occurrence (low=blue, high=red). The plots also show the locations of the haul outs (black points) and the number represents the seal tag ID.
